# Association of sleep duration and insulin resistance in Taiwanese vegetarians

**DOI:** 10.1186/1471-2458-12-666

**Published:** 2012-08-16

**Authors:** Jiunn-Kae Chang, Malcolm Koo, Vivia Yu-Ying Kao, Jui-Kun Chiang

**Affiliations:** 1 Department of Biotechnology, Chia Nan University of Pharmacy & Science, Tainan, Taiwan; 2 Dalla Lana School of Public Health, University of Toronto, Ontario, Canada; 3 Department of Family Medicine, Buddhist Dalin Tzu Chi General Hospital, 2 Min-Sheng Road, Dalin, Chiayi, Taiwan

**Keywords:** Sleep duration, Insulin resistance, Vegetarian

## Abstract

**Background:**

Short sleep duration has been reported to associate with increased insulin resistance. However, no studies have investigated whether such association exists in vegetarians. The aim of this study was to investigate the association between sleep duration and insulin resistance in Taiwanese vegetarians.

**Methods:**

A total of 1290 individuals were recruited from a regional hospital in south Taiwan during their regular routine physical examination. Only individuals who described themselves as Buddhist vegetarians were included in the study. Demographic information and clinical characteristics were collected and multiple logistic regression analysis was used to evaluate the association between sleep duration and insulin resistance.

**Results:**

A total of 433 vegetarians were included in the study. Results from univariate logistic regression indicated that insulin resistance was significantly associated with male sex, greater waist circumference, higher triglyceride levels, lower high-density lipoprotein cholesterol levels, higher plasma creatinine levels, higher alanine transaminase levels, greater energy expenditure, and sleep duration of more than 8 hours per night. Multiple logistic regression revealed that insulin resistance was significantly and independently associated with sleep duration of more than 8 hours per night (odd ratios = 2.27, 95% confidence interval = 1.24, 4.11) after adjusting for waist circumference and levels of alanine transaminase.

**Conclusions:**

Sleep duration of more than 8 hours per night is an independent risk factor associated with increased insulin resistance in vegetarians.

## Background

The marked reduction in average sleep duration in modern society has been suggested as one of the risk factors responsible for the increase in prevalence of metabolic syndrome, diabetes, and obesity [1-3]. Short sleep duration has been shown to independently associate with weight gain [4] and an increased risk of hypertension [5], cardiovascular disease [6], and colorectal adenoma [7]. In addition, a U-shaped relationship between sleep duration and hyperglycemia [8], metabolic syndrome [9], diabetes [10,11], and even mortality [12] has been reported. Recently, an association between short sleep duration and increased insulin resistance was noted [13,14]. It has been postulated that decreased sleep duration could elevate appetite, increase body weight, decrease glucose tolerance, increase blood pressure, and increase sympathetic tone [15,16]. Insulin resistance is defined as the decreased tissue response to insulin-mediated cellular actions. It is the main pathophysiological feature of the metabolic syndrome, which is strongly associated with a number of health problems such as diabetes, cardiovascular disease, hypertension, and hyperlipidaemia [17-20].

A few studies have reported the beneficial effects of vegetarian dietary patterns on the prevention of metabolic syndrome. Foods with a low glycemic index and high dietary fiber content typically consumed by vegetarians are associated with a lesser increase in post-prandial blood glucose and insulin secretion [21]. In a cross-sectional survey of a representative sample of 19,003 adults in a suburban area of Beijing, vegetarians had a lower risk of metabolic syndrome [22]. Similar results where a vegetarian dietary pattern is associating with a lower risk of metabolic syndrome were also observed in a cross-sectional study of 773 adults randomly selected from the Adventist Health Study 2. The relationship persisted after controlling for the possible confounding effects of age, sex, ethnicity, smoking, alcohol intake, physical activity, and dietary energy intake [23]. In addition, a study on female Taiwanese reported that serum total cholesterol, LDL-cholesterol, triglycerides, fasting blood glucose, and blood pressure were significantly lower in long-term vegetarians compared with omnivores [24]. However, it is not known whether the association between sleep duration and insulin resistance also exists in vegetarians who are potentially less prone to insulin resistance compared to omnivores. The aims of this study were to investigate the association between sleep duration and insulin resistance in Taiwanese vegetarians and examine whether such association is independent of adiposity. The area under the receiver operating characteristic curve was used to quantify prediction accuracy.

## Methods

### Study participants

Participants were recruited from a regional hospital in south Taiwan at the time of their regular routine physical examination between May 2007 and April 2009. Demographic information and clinical characteristics including age, sex, body weight, height, waist circumference, a family history of diabetes or hypertension, dietary habit, sleep duration, and sitting blood pressure were collected. Individuals with diabetes, cancers, and chronic renal failure on hemodialysis were excluded from the study.

In addition, only individuals who described themselves as Buddhist vegetarians were included in the study. Chinese Buddhist vegetarians avoid all meat and fish consumption in their diet. Other than that, their diet is similar to the usual Chinese diet in terms of cooking methods, choice of staple (primarily rice), and consumption of fruits and vegetables. Some Chinese Buddhist vegetarians also include eggs and dairy products in their diets [25]. Results from a nutritional study on 109 Taiwanese vegetarians and 107 age- and sex-matched omnivores found that energy intake was significantly lower in female vegetarians but not in male vegetarians when compared with the omnivore counterparts. Vegetarians also consumed significantly less protein, fat, and cholesterol than the omnivores [26]. Another study on 49 healthy Buddhist lactovegetarians and 49 omnivores in Taiwan also reported that vegetarians consumed less energy, fat, and protein, but more dietary fiber than the omnivores [27].

Body weight and height were measured while all participants were minimally clothed without shoes using digital scales. Waist circumference was obtained at the umbilical level using a measuring tape. Body mass index was computed as the ratio of weight (in kilograms) to height (in meters) squared.

Sleep duration and energy expenditure were assessed using a questionnaire administered face-to-face by a research assistant. Mean sleep duration was calculated as [(5/7 × weekday sleep duration) + (2/7 × weekend sleep duration)] [28]. Daily energy expenditure was estimated using a seven-day physical activity recall questionnaire (Chinese version) [29]. The study protocol was approved by the Research Ethics Committee of the Buddhist Dalin Tzu Chi General Hospital (Approved No. B09702038, B09604003-1) and all participants provided written informed consent.

### Sample processing and analyses

Total cholesterol, triglyceride, high-density lipoprotein cholesterol (HDL-C), low-density lipoprotein cholesterol (LDL-C), glucose, white blood cell counts, blood urea nitrogen (BUN), creatinine, insulin, and alanine transaminase (ALT) were measured using blood sample collected from each participants after a minimum eight hour fasting period. The tests were analyzed using an auto-analyzer (Sysmex XE-2100 Blood Cell Analyzer, Kobe, Japan) at the central laboratory of the study hospital. Plasma fasting insulin concentrations were measured using human insulin Enzyme Linked Immunosorbent Assay (ELISA) kit (BioSource Europe S.A., Nivelles, Belgium). Insulin resistance was defined by homeostasis model assessment for insulin resistance (HOMA-IR). HOMA-IR was calculated by dividing the product of fasting plasma glucose (mg/dL) and fasting plasma insulin (mU/L) by 405 [30]. Insulin resistance was defined as the value of HOMA-IR of highest quartile of its empirical distribution [31], which is ≥ 2 in this study.

### Statistical analysis

Sleep duration was dichotomized using a cut off value of 8 hours or less in all analyses. Continuous variables were expressed as mean ± standard deviation (SD). Categorical variables were represented by frequency and percentage. In the univariate analysis, Student *t*-tests or Wilcoxon rank-sum tests were used to compare differences between the two groups, depending on the results of Shapiro-Wilk test of normality. Fisher’s exact tests were used to compare the categorical variables between the two groups.

Univariate logistic regression analysis was performed for the variables on demographic and clinical characteristics with the binary variable insulin resistance as the dependent variable. Multiple logistic regression analysis with stepwise variable selection procedure using the Akaike’s Information Criterion (AIC) was conducted to compute adjusted odds ratios (ORs) and 95% confidence intervals (CI)s for the association of sleep duration with insulin resistance. Variables that were under evaluation during the stepwise model development included sex, age, waist circumference, white blood cell count, total cholesterol, triglyceride, HDL-C, LDL-C, creatinine, blood urea nitrogen, ALT, education level, energy expenditure, and sleep duration. Hosmer and Lemeshow Goodness-of-Fit test was conducted to determine the adequacy of the fitted logistic models.

A receiver operating characteristic (ROC) analysis was conducted to evaluate the ability for correctly discriminating the study participants of low and elevated HOMA-IR. Plots of the sensitivity (true-positive fraction) versus 1 - specificity (false-positive fraction) were made and the overall diagnostic accuracy was quantified using the area under the curve (AUC). All analyses were performed using the statistics package R 2.13.0 [32] and a 2-sided P < 0.05 was considered statistically significant.

## Results

A total of 1,290 individuals were approached at their physical examination. Of those, 515 were identified as vegetarians and 485 (94.2%) agreed to participate in the study. A further 35 individuals were excluded because of their diseases and 17 individuals had missing data. The basic characteristics of the 433 study participants are shown in Table 1. Individuals with sleep duration of more than 8 hours had significantly greater waist circumference, energy expenditure, white blood cell count, triglyceride, plasma creatinine, fasting glucose, insulin, and HOMA-IR but lower HDL-C than those with sleep duration of 8 hours or less. Male participants and those with education levels of high school and above were also significantly associated with sleep duration of more than 8 hours.

**Table 1:** Characteristics of the study participants according to sleep duration

**Variable**	**Sleep duration**		**P**
	**≤ 8 hours,n = 371**	**> 8 hours,n = 62**	
Sex			<0.001
Male	77 (20.8)	30 (48.4)	
Female	294 (79.2)	32 (51.6)	
Age (years)	55.9 ± 9.0	63.7 ± 8.3	0.079
Body mass index (kg/m^2^)	22.9 ± 2.8	23.2 ± 2.3	0.238
Waist circumference (cm)	74.0 ± 7.8	77.2 ± 7.9	0.002
Systolic blood pressure (mmHg)	125.3 ± 19.1	124.2 ± 19.7	0.914
Education			0.033
Primary school and below	112 (32.1)	11 (18.3)	
High school and above	237 (67.9)	49 (81.7)	
Alcohol use	4 (1.1)	0	1.000
Smoking	1 (0.3)	0	1.000
Energy expenditure (Kcal × 10^3^/day)	2.7 ± 1.0	3.2 ± 1.3	0.034
Family history of hypertension	58 (15.6)	8 (12.9)	0.704
Family history of diabetes	90 (24.3)	17 (27.4)	0.634
White blood cell count (×10^3^/μL)	6.0 ± 1.6	6.6 ± 1.6	0.014
Total cholesterol (mmol/L)	4.6 ± 0.8	4.5 ± 0.7	0.303
Triglyceride (mmol/L)	1.2 ± 0.7	1.5 ± 0.8	0.001
HDL-C (mmol/L)	1.42 ± 0.36	1.18 ± 0.25	<0.001
LDL-C (mmol/L)	2.98 ± 0.74	2.95 ± 0.60	0.902
ALT (IU/L)	22.1 ± 18.6	25.4 ± 17.8	0.080
Blood urea nitrogen (mmol/L)	4.7 ± 1.3	4.4 ± 1.2	0.393
Creatinine (μmol/L)	56.3 ± 17.7	60.7 ± 16.2	0.013
Fasting glucose (mmol/L)	4.88 ± 0.49	5.02 ± 0.47	0.037
Insulin (mU/L)	6.2 ± 5.7	7.9 ± 6.4	0.020
HOMA-IR	1.4 ± 1.3	1.8 ± 1.4	0.010

Continuous and categorical variables were expressed as mean ± standard deviation and number (percentage).

HDL-C: high-density lipoprotein cholesterol.

LDL-C: low-density lipoprotein cholesterol.

ALT: alanine transaminase.

**Figure 1:**
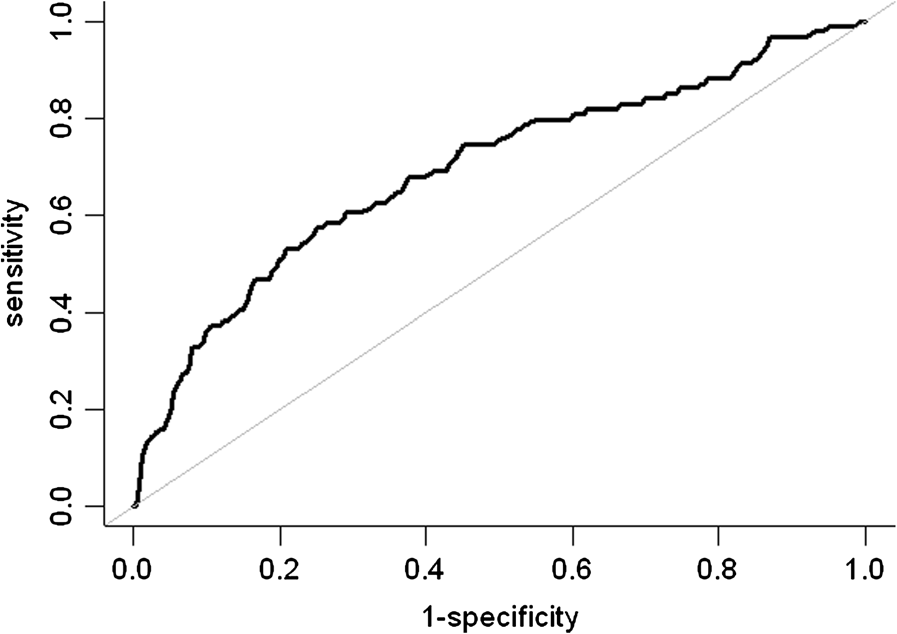
**The receiver operating characteristics curve.** Model was obtained from the multivariate logistic regression with the area under the curve = 0.69.

Univariate logistic regression analysis revealed that females, greater waist circumference, higher triglycerides, higher creatinine, higher ALT, higher energy expenditure, lower HDL-C, and sleep duration of more than 8 hours were associated with increased risk of insulin resistance (Table 2). Table 3 shows the final model for the predictors in the multivariate logistic regression analysis. The risk of insulin resistance in individuals with sleep duration of more than 8 hours was 2.3 times higher than those with shorter sleep duration, after adjusted for waist circumference and ALT level. The Hosmer-Lemeshow test showed a good fit (P = 0.259). The AUC for the prediction model was 0.69 (Figure 1). The programming code for calculating the probability of insulin resistance based on the final model is provided in Additional file 1: Appendix 1.

**Table 2:** Results of univariate logistic regression associated with insulin resistance

	**β**	**Standard Error**	**P**	**Odds Ratio (95% Confidence Interval)**
Sex (male vs. female)	−1.51	0.14	<0.001	2.20 (1.34-3.59)
Age	−0.01	0.01	0.928	1.00 (0.97-1.02)
Waist circumference (cm)	0.07	0.01	<0.001	1.07 (1.04-1.11)
White blood cell count (×10^3^/μL)	0.13	0.04	0.066	1.14 (0.99-1.31)
Total cholesterol (mmol/L)	−0.13	0.14	0.347	0.87 (0.66-1.15)
Triglyceride (mmol/L)	0.35	0.14	0.016	1.42 (1.07-1.89)
HDL-C (mmol/L)	−1.25	0.38	0.001	0.29 (0.13-0.59)
LDL-C (mmol/L)	−0.09	0.16	0.595	0.92 (0.66-1.26)
Creatinine (μmol/L)	0.02	0.01	0.005	1.02 (1.01-1.03)
Blood urea nitrogen (mmol/L)	0.09	0.09	0.315	1.09 (0.92-1.29)
ALT (IU/L)	0.02	0.01	0.002	1.02 (1.01-1.04)
Education (primary school and below vs. above)	−0.40	0.28	0.153	0.67 (0.38-1.14)
Energy expenditure (Kcal × 10^3^/day)	0.28	0.10	0.008	1.32 (1.07-1.62)
Sleep duration (>8 hrs vs. ≤8 hrs)	1.00	0.29	0.001	2.72 (1.52-4.80)

Reference category for education was primary school and below.

HDL-C: high-density lipoprotein cholesterol.

LDL-C: low-density lipoprotein cholesterol.

ALT: alanine transaminase.

**Table 3:** Results of multiple logistic regression analysis associated with insulin resistance

	**β**	**Standard Error**	**P**	**Odds Ratio (95% Confidence Interval)**
Intercept	−6.43	1.17	<0.001	
Sleep duration (>8 hrs vs. ≤8 hrs)	0.82	0.31	0.007	2.27 (1.24-4.11)
Waist circumference (cm)	0.06	0.02	<0.001	1.06 (1.03-1.10)
ALT (IU/L)	0.01	0.01	0.023	1.01 (1.00-1.03)

ALT: alanine transaminase.

Variables eliminated during the stepwise regression process included sex, age, white blood cell count, total cholesterol, triglyceride, high-density lipoprotein cholesterol, low-density lipoprotein cholesterol, creatinine, blood urea nitrogen, education level, and energy expenditure.

Akaike’s Information Criterion (AIC): 423.5.

The Hosmer-Lemeshow test, P = 0.259.

Nagelkerke R^2^ = 0.13.

**Figure 2:**
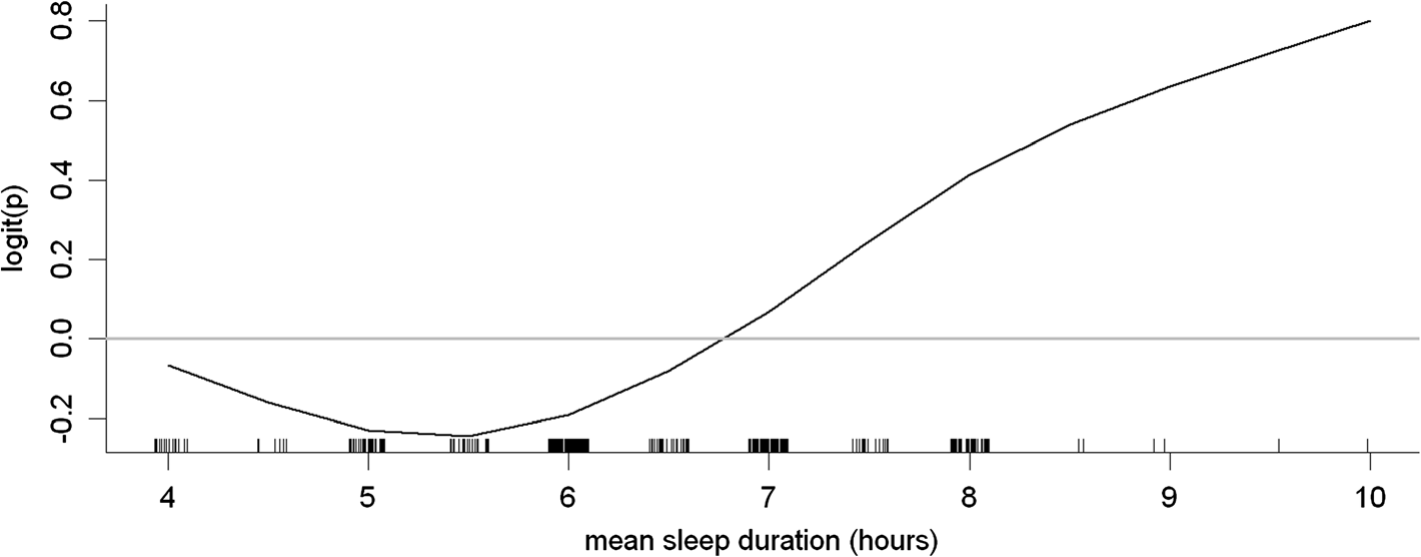
**Plot of nonparametric smoothing from generalized additive model of sleep.** Duration after adjusting for waist circumference and alanine transaminase levels. The y-axis is logit(p) where p is the probability of insulin resistance.

An additional multivariate logistic regression analysis was conducted with tertiles of sleep duration. With adjustment of waist circumference and ALT level, short sleep duration (< 6 hours), compared to sleep duration of 6 to 8 hours, was not significantly associated with insulin resistance (P = 0.721). However, long sleep duration (> 8 hours), compared to sleep duration of 6 to 8 hours, was significantly associated with insulin resistance (P = 0.012). Therefore, a U-shaped pattern between sleep duration and insulin resistance was not observed in the present study. The choice of cutpoints for sleep duration was based on a plot of nonparametric smoothing obtained from the generalized additive model of sleep duration (Figure 2).

## Discussion

The present study, to the best of our knowledge, is the first to report that sleep duration of 8 hours or more is associated with increased risk of insulin resistance in vegetarians. The association remained significant after adjustment for waist circumference and liver function. Previous reports on sleep duration and insulin resistance or metabolic syndrome have indicated the existence of either an inverse [13,14] or a U-shaped relationship [9]. Short sleep duration has been suggested to associate with an increase risk of metabolic syndrome through an upregulation of the neuroendocrine control of appetite, in particular, reduced leptin and increased ghrelin levels. These endocrine changes could lead to weight gain and a higher risk of obesity [33]. Short sleep duration can increase cortisol concentrations and sympathetic nervous system activity, which are associated with increased risk of developing insulin resistance [34].

On the other hand, there is no clear mechanism to explain the association between long sleep duration and insulin resistance. It has been hypothesized that long sleep duration is an indication of the presence of comorbidity such as depression [35]. In addition, low level of physical activity [36], and low socioeconomic status [35] have been reported to associate with long duration of sleep. In the present study, education levels and energy expenditure were not significantly associated with long sleep duration. Another possible mechanism for linking sleep duration with insulin resistance is through the mediation of cytokines. It has been shown that each additional hour of habitual sleep duration was significantly associated with an 8% increase in C-reactive protein (CRP) levels and 7% increase in interleukin-6 (IL-6) levels [37]. Chronic elevations in these proinflammatory cytokines have been shown to associate with an increased risk of diabetes [28] and therefore, habitual long sleep duration could alter the regulation of these cytokines and potentially affect glucose metabolism. Moreover, it is possible that long sleep duration might be a consequence of another unrecognized causative risk factor for insulin resistance. In addition, whether the lack of association between short sleep duration and insulin resistance was the results of a protective effect of vegetarian dietary patterns will need further investigations.

In addition to sleep duration, greater waist circumference and elevated levels of ALT were associated with insulin resistance. Waist circumference is considered as the best anthropometric indicator of abdominal visceral obesity [38] and has been used as a non-invasive method of identifying the presence of insulin resistance [39-41]. ALT, found primarily in the liver, is a specific indicator of heptocellular health. Several prospective studies have demonstrated elevated levels of ALT were an independent predictor of type 2 diabetes [42,43].

This study has some limitations that need to be considered. First, our study used cross-sectional design and therefore precluding direct inferences concerning temporal relationship of sleep duration and insulin resistance. Nevertheless, available evidence in diabetic studies indicated that disturbed sleep can impair glucose metabolism and poorly controlled glucose levels can also impair sleep [44]. Second, we have only measured sleep duration but not sleep quality. It is possible that poor sleep quality, sleep apnea syndrome [45], and depression [46] can impact on insulin resistance. A more detailed questionnaire regarding sleep quality should be included in future studies. Third, information about sleep duration was based on participants’ self-report. Nevertheless, self-report have been shown to be a valid measurement of speed duration compared with polysomnography [47].

## Conclusions

In conclusion, this study showed that longer duration of sleep was independently associated with insulin resistance in vegetarians, after controlling for potential confounders. Clinicians can use the codes provided in Additional file 1: Appendix 1 and readily available measurements of sleep duration, waist circumference, and ALT level as a simple adjunctive method for early detection of insulin resistance.

## Abbreviations

AIC: Akaike’s Information Criterion; ALT: Alanine transaminase; AUC: Area under the curve; BUN: Blood urea nitrogen; ELISA: Enzyme Linked Immunosorbent Assay; HDL-C: High-density lipoprotein cholesterol; HOMA-IR: Homeostasis model assessment for insulin resistance; LDL-C: Low-density lipoprotein cholesterol; ROC: Receiver operating characteristic.

## Competing interests

The authors declare that they have no competing interests.

## Authors’ contributions

JK Chiang conceived the research questions, designed the study and drafted the initial manuscript. M Koo was involved in the interpretation of the analysis and revision of the manuscript. JK Chang was involved in data analysis. YY Kao was involved in preparatory field works. All authors approve the final manuscript.

Institution at which the work was performed: Buddhist Dalin Tzu Chi General Hospital, Chiayi, Taiwan.

Grand support: Buddhist Dalin Tzu Chi General Hospital, Chiayi, Taiwan.

## Pre-publication history

The pre-publication history for this paper can be accessed here:

http://www.biomedcentral.com/1471-2458/12/666/prepub

## Supplementary Material

Additional file 1 **Appendix 1. **Programming code in OpenOffice Calc, Microsoft Excel, and R for calculating the probability of elevated insulin resistance (HOMA-IR >2) based on the multiple logistic regression. (DOC 23 kb) Click here for file
