# Supplementary material for: Association of sleep duration and insulin resistance in Taiwanese vegetarians
Source: BMC Public Health. 2012 Aug 16;12:666. doi: 10.1186/1471-2458-12-666 (PMC3490851; doi:10.1186/1471-2458-12-666)
Supplement: Additional file 1 — Appendix 1. Programming code in OpenOffice Calc, Microsoft Excel, and R for calculating the probability of elevated insulin resistance (HOMA-IR >2) based on the multiple logistic regression. (DOC 23 kb) [file 1471-2458-12-666-S1.doc]

**Appendix**:

Programming code in OpenOffice Calc, Microsoft Excel, and R for calculating the probability of elevated insulin resistance (HOMA-IR >2) based on the multiple logistic regression.

1. In OpenOffice Calc or Microsoft Excel: Key in the values for sleep duration (>8 hours =1, ≤8 hours =0) in the A1cell, waist circumference (cm) in the A2 cell, and alanine transaminase (ALT) (IU/L) in the A3 cell.

Key in the following formula in any empty cell on the spreadsheet to obtain the probability of insulin resistance.

= 1/EXP(-(-6.43 + 0.82*A1 + 0.06*A2 + 0.01*A3)+1)

1. In R environment

To calculate the probability of insulin resistance, substitute the values for the variables X_1_ to X_3_ in the following regression equation.

yhat<- (-6.43 #constant

+ 0.82*X1 #X1 = sleep duration (>8 hours =1, ≤ 8 hours =0

+ 0.06 *X2 #X2 = waist (in cm)

+0.01*X3 #ALT (IU/L)

)

phat <- 1/(exp(-(yhat))+1)

phat #copy these syntax and past on the R console, and press enter
